# Prospective Randomized Study Comparing the External Fixator and Volar Locking Plate in Intraarticular Distal Radius Fractures: Which Is Better?

**DOI:** 10.7759/cureus.6849

**Published:** 2020-02-02

**Authors:** Aryan Sharma, Subodh Pathak, Harinder Sandhu, Priyank Bagtharia, Naveen Kumar, Rajdeep S Bajwa, Vineet Pruthi, Jasneet S Chawla

**Affiliations:** 1 Orthopedics, Maharishi Markandeshwar Institute of Medical Sciences and Research. Maharishi Markandeshwar (deemed to be University), Ambala, IND

**Keywords:** intraarticular fracture distal radius, volar plating, external fixator, modified green & o’brien score

## Abstract

Introduction

Various treatment options for patients with an intraarticular distal radius fracture are available, but in cases of comminuted fractures, these narrow down to either a volar locking plate, an external fixator, or a combination of these two. We conducted this prospective study to compare the external fixation and internal fixation of intraarticular fractures of the distal radius in terms of clinical/functional outcome and complications and with the available literature.

Material and method

This prospective randomized study consisted of a total number of 30 patients with intraarticular fractures of the distal end of the radius divided randomly into two groups (A and B), treated by external fixation (Group A) and volar plating (Group B), in a tertiary care institute during the study period.

Result

The most patients were males >50 years of age, with injury to the right dominant hand most commonly caused by a fall on an outstretched hand. As per the modified Green & O’Brien scoring system, the volar plating group showed the final result as excellent in two (13.33%), good in seven (46.6%), fair in four (26.6%), and poor in two (13.3%) whereas an excellent outcome was seen in one (6.66%), good and fair in five patients each (33.3%), and poor in four (26.66%) patients at the six months follow-up.

Conclusion

Overall, both fixation techniques seem to apply sufficient stabilization to restore function and retain anatomy; however, volar locking plates have certain advantages over external fixator in the early postop period in terms of earlier recovery and mobilization.

## Introduction

The management of distal radial fractures has progressed in the past three decades from the use of a cast application to a number of operative modalities such as closed reduction and external fixation or open reduction and fixation with locking plates [1]. Along with sex, age group, ethnicity, family history, and early menopause, decreased bone mineral density also accounts as one of the risk factors for fractures of the distal end of the radius [2]. Intraarticular fractures at the distal radius are complex and unstable fractures generally resulting due to high-energy trauma. Understanding both the pathology and mechanics of these injuries highlights many issues faced by patients, including pain, swelling, weakness, with limitation of movements and joint arthritis, along with instability faced in cases where the anatomical reduction of fragments and associated ligaments are not achieved [3]. The chances of unfavorable outcomes after an intraarticular fracture of the distal radius increase with the occurrence of malunion and stiffness of the wrist joint; surgical corrections are often needed to achieve a functionally acceptable outcome and anatomical position [4]. Of the two modes of treatment, conservative and surgical, generally, closed reduction is the preferred method of treatment in a stable fracture but surgical interventions are required for an unstable fracture with intraarticular displacement [2]. Precise restoration of the distal end radius articular surface is a crucial factor in regulating a successful outcome, as there is a strong correlation between osteoarthritis and residual articular congruity [5]. Closed reduction followed by fixation with a bridging external fixator has been employed in unstable distal radial fractures for a long time, with adequate functional outcomes. The rationale in favor of employing the external fixator technique includes continuation in fracture reduction under fluoroscopic view, augmentation in reduction by means of ligamentotaxis, along with the ability to preserve reduction until fracture healing takes place. The benefits of an external fixator are the comparative easy application of hardware, minimal operative exposure required, and decreased operative trauma [1]. Internal fixation, however, gives better results in terms of reduction and can significantly decrease total surgical complications, as well as infection due to the pin track, whereas the external fixator reduces surgical time and decreases trauma due to surgery [6]. In addition, internal fixation allows greater stability and a shorter rehabilitation time; functional outcomes are seen better with a volar locking plate in comparison with an external fixator. External fixators are bulky, cause inconvenience for the patients, and are, therefore, usually reserved for more severe fractures [7]. Various studies have previously compared the merits and demerits of external fixation with internal fixation, but there is a lack of sufficient evidence about which technique has the best possible outcome in elderly patients [4].

Therefore, the purpose of our study is to compare external fixation and internal fixation in terms of clinical/functional/radiological outcomes and complications in cases with an intraarticular distal end of radius fracture.

## Materials and methods

This prospective study was conducted on 30 patients, of either gender, presenting with intraarticular fractures of the distal end of the radius in tertiary care center after due clearance from the institutional ethics committee.

Upon admission, patients were subjected to evaluation with detailed history, relevant investigations, and thorough clinical examination, and they received primary care. Fractures were classified as per the Arbeitsgemeinschaft für Osteosynthesefragen / Association of the Study of Internal Fixation (AO/ASIF) classification system. Cases selected per the inclusion and exclusion criteria (Table 1) were randomized into two groups, Group A (external fixation) and Group B (internal fixation) of 15 each using random number tables generated online.

**Table 1:** Criteria for case selection AO: Arbeitsgemeinschaft für Osteosynthesefragen

Inclusion Criteria	Exclusion Criteria
Intraarticular fractures distal radius AO type B and C	Open fractures
Age 18-80 yrs	Bilateral radius fractures
Unilateral radius fractures	Head injury/spinal injury
Polytrauma cases	Previous fracture in the same limb
	Pathological fractures
	Injury >2 wks old
	Refusal to participate/cooperate

The external fixation group underwent a closed reduction of the fracture under fluoroscopy followed by the application of a wrist-spanning external fixator supplemented with or without K wires if necessary. The internal fixation group underwent open reduction and internal fixation of the fracture by a volar locking plate by a modified Henry’s approach. In two patients, the volar plate was supplemented with a K wire for distal radial ulnar joint instability, which was removed at six weeks.

Follow-up evaluation

All the participating cases were followed up at four weeks, six weeks, and 12 weeks until a minimum of six months postop. By postop one week, patients treated with the external fixator began finger and grip strength exercises. A removable plaster of Paris (POP) splint was applied to the volar plating group postoperatively and started on range of motion (ROM) and grip strength exercises as per the tolerance of the patient. Bone healing was determined radiographically by the appearance of bridging trabeculae across the fracture and clinically by the fracture site being non-tender to palpation [8]. The fixator was removed at a mean interval of 7.2 weeks (6 weeks to 8 weeks). ROM measurements were done using the goniometer, and grip strength measurements were done using the Jamar dynamometer at level 2 by an occupational therapist. Patients were evaluated for criteria such as pain, grip power, ROM around the wrist joint and activity and then scored according to the modified Green & O’Brien scoring system at three months and six months postop [1]. Interpretation of scoring was done by two authors (PB and RB) who were blinded with patient details. Scores less than 65 were interpreted as poor, 65 to 79 as fair, 80 to 89 as good, and above 90 to 100 were considered excellent.

Statistical analysis

Data were described in terms of range, mean±standard deviation (±SD), frequencies (number of cases), and relative frequencies (percentages) as appropriate. A comparison of quantitative variables between the study groups was done using the student t-test. For comparing categorical data, the chi-square (χ2) test was performed and the exact test was used when the expected frequency is less than 5. A p-value of less than 0.05 was considered statistically significant.

## Results

**Figure 1:**
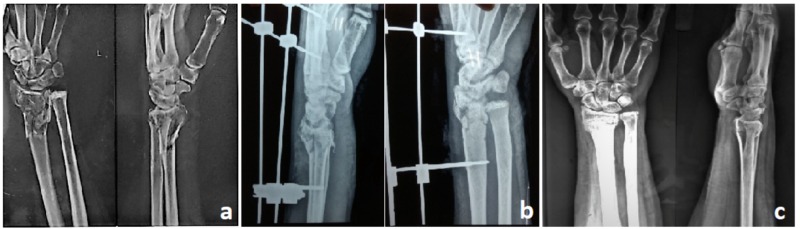
A: Preoperative radiograph showing intraarticular distal radius fracture (AO type C3); B: Postoperative radiograph with external fixator in situ; C: Six-month follow-up radiograph showing radiological union

The mean age of patients was 54.03±10.52 SD (range 24-78 years) and the maximum number of cases belonging to >50 yrs of age group. Males were predominantly affected in our study with 18 (60%) of patients being male and 12 (40%) being female. Overall, the male to female ratio was 1.5:1 in our study (p-value= 0.456). Fall on an outstretched hand (56.66%) was the most common cause of injury in this study, followed by roadside accidents (33.3%). Right-handed injury 19 (63.3%) was most common in our study (p-value=0.021) and the dominant side was involved in 19 (63.3%) of total patients (p-value=0.447). Associated injuries were seen in six (20%) of the patients, most common being the proximal tibia fracture (two; 6.6%). In the present study, it was observed that the maximum number of patients had a C2 fracture (seven; 23.3%) followed by B3 (six; 20%), C1 and B1 (five; 16.66%) cases each while three patients (10%) had C3 fracture (Figures 1A-1C).

**Figure 2:**
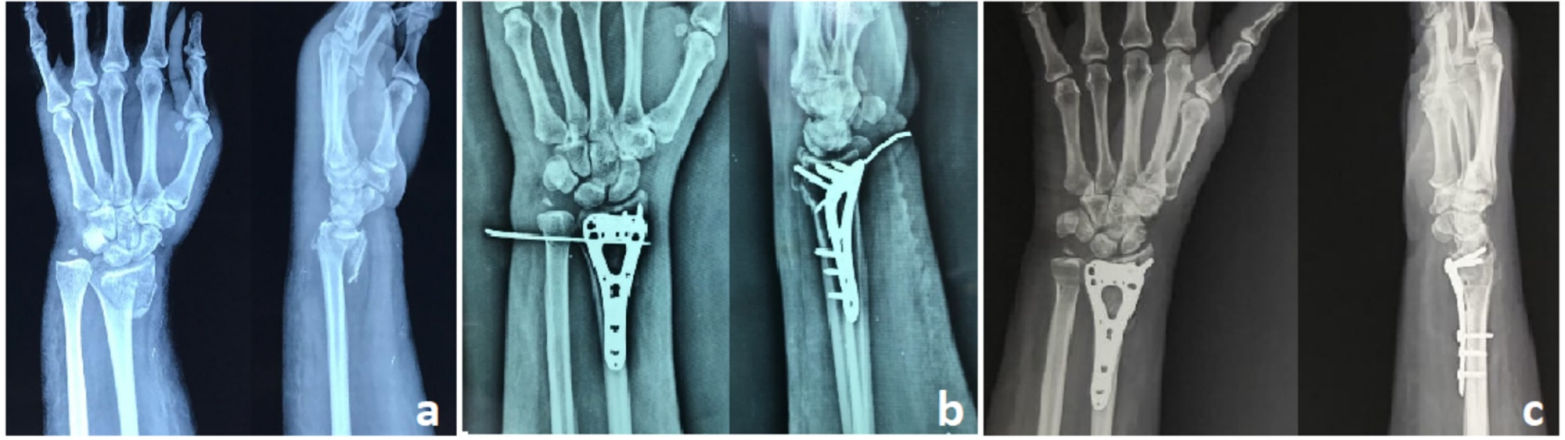
A: Preoperative radiograph showing intraarticular distal radius and ulnar styloid fracture; B: Postoperative radiograph with the volar locking plate and DRUJ K wire in situ; C: Six-month follow-up radiograph showing radiological union DRUJ: Distal radioulnar joint; K wire: Kirschner wires

The mean duration of surgery was 68.4 min in the volar plating group and 51.13 min in the external fixator group (p-value=0.000001). Mean time of clinical union was 7.4 weeks in the volar plating group and 6.65 weeks in the external fixator group (p-value=0.222) (Figures 2A-2C).

Wrist stiffness (five; 16.66%) was the most common complication encountered in our study, five (13.33%) in the external fixator group and one (3.33%) in the plating group followed by pin tract infection in one external fixator patient and superficial wound infection in one volar plating patient.

Mean range of motion was evaluated in both groups at three months and six months follow-up and the difference was statistically significant (Table 2).

**Table 2:** Range of motion at three-month and six-month follow-up * significant values Exfix: external fixator; ROM: range of motion; PF: palmar flexion; DF: dorsiflexion; Sup: supination; Pro: Pronation; UD: ulnar deviation; RD: radial deviation

ROM	At 3 months			At 6 months		
	Exfix	Plating	p-value	ExFix	Plating	p-value
PF	54.73±5.90	64.87±4.70	0.000016*	63.13±2.72	75.53±6.09	0.000000*
DF	48.80±4.75	60.20±4.77	0.000000*	60.27±1.98	67.93±3.69	0.000000*
Sup	59.80±4.95	67.33±4.64	0.000186*	69.27±2.76	76.60±4.85	0.000022*
Pro	57.47±4.56	65.67±3.90	0.000013*	66.67±2.66	74.87±3.68	0.000000*
UD	18.40±2.29	23.53±1.85	0.000000*	25.40±2.64	28.60±3.18	0.005641*
RD	11.53±1.60	14.53±1.36	0.000006*	14.60±1.06	17.27±1.62	0.000011*

Radiological parameters were restored in both the groups and the difference was statistically insignificant (Table 3).

**Table 3:** Radiological parameters at the six-month follow-up

	Plating		ExFix		p-value
	Mean±SD	Range	Mean±SD	Range	
Volar tilt (degree)	5.20±4.63	-2° to 12°	3.20±5.02	-4° to 12°	0.266
Radial shortening (mm)	2.17±1.13	0.5-4 mm	2.27±0.82	1-3.5 mm	0.783
Radial inclination (degree)	19.23±2.54	14°-22°	17.90±2.48	14°-22°	0.157

**Figure 3:**
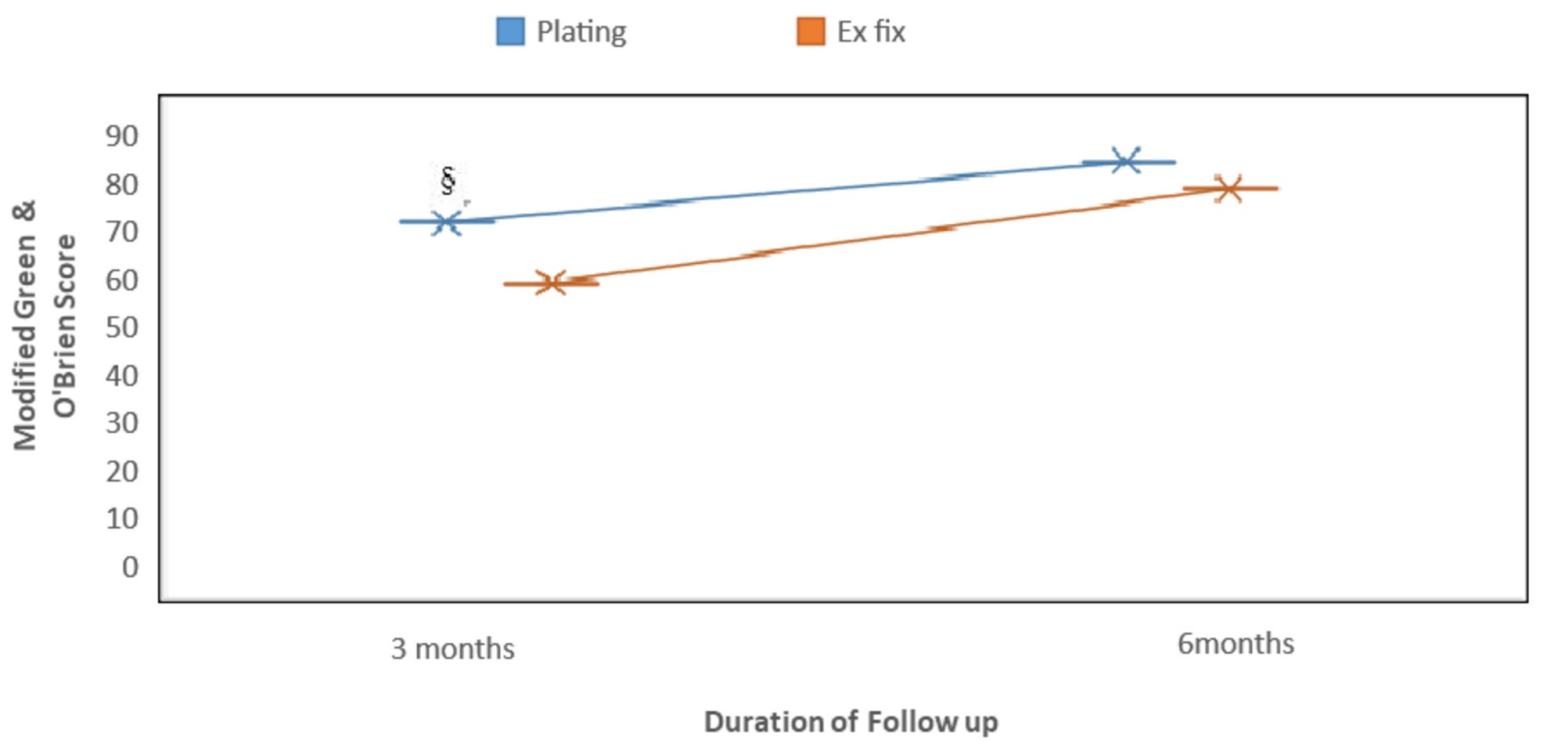
Graph showing modified Green & O’Brien scores at different follow-ups Statistical significance is seen at three months although no difference is noted at the six-month follow-up. § Mean statistical significance (p=0.016)

In our study, as per the modified Green & O’Brien scoring system, the volar plating group showed the final result as excellent (two; 13.33%), good (seven; 46.6%), fair (four; 26.6%), and poor (two; 13.3%) whereas the external fixator group had the final result at six months' follow-up as excellent (one; 6.66%), good (five; 33.3%), fair (five; 33.3%), and poor (four; 26.66%). The total score was 68 in the plating group and 57.33 in the fixator group at three months, which was statistically significant (p-value 0.016) and improved during the course of physiotherapy to 79 in the plating group and 74.33 in the fixator group, and the difference became statistically non-significant (p-value 0.253) by the six months; follow-up (Figure 3).

A subanalysis of the data also showed that the external fixator group performed comparable and somewhat better than the plating group in the management of complete intraarticular fractures, and extensively comminuted C3 patients performed satisfactorily when managed with closed reduction and external fixation, as shown in Table 4.

**Table 4:** Final outcome in AO type C (complete intraarticular) fracture AO: Arbeitsgemeinschaft für Osteosynthesefragen; Exfix: external fixator

	Number of cases at final 6 months follow up as per AO type of fracture			
AO type	Excellent	Good	Fair	Poor
C1		1 Exfix 2 Plating	1 Plating	1 Exfix
C2	1 Exfix	2 Plating	2 Plating	1 Exfix 1 Plating
C3		1 Exfix	1 Exfix	1 Plating

## Discussion

In the past 100 years, most distal radius fractures have been treated conservatively. However, the results of several studies have demonstrated that the satisfactory outcome of such conservative treatment is not consistent and there are a lot of controversies on whether the anatomical reduction of distal radial fractures is essential but there is no controversy that maintaining satisfactory reduction is often difficult by a simple plaster cast. Trumble et al. stated that the degree to which articular step-off, gapping between fragments, and radial shortening can be improved with surgery correlate strongly with improved outcome [9]. Today, several different surgical strategies, such as external fixations, percutaneous pinning, or open reduction and internal fixation are available.

The open reduction and internal fixation of a fracture are usually done via a volar or sometimes a dorsal or combined approach. However, this technique of fixation is technically demanding and is preferred only by surgeons with sufficient skill sets. On the other hand, external fixation has been employed for the past 80 years with various configurations available to suit the pattern of injury and the experience of the surgeon. External fixation often requires supplementation with K wires, bone grafting, or stabilization of the radioulnar joint to assist in and maintain the reduction in complex fractures [10]. Arthroscopic-assisted reduction during external fixation enhances the ability to achieve satisfactory reduction due to the direct visualization of the joint surface and concomitant assessment of the wrist ligaments. In recent times, with the advent of lower-profile polyaxial locking screw implants, it has become easier to address fractures with extensive fragmentation, variations in normal anatomy, and dorsal metaphyseal instability with a single surface implant leading to the increased popularity of the open reduction and internal fixation of these fractures among surgeons [11-12].

Although some studies have shown good results for various methods, the choice of the best option still remains controversial, as prospective randomized studies have not shown convincingly superior results for any of the procedures.

Chung KC et al. concluded in their study on the treatment of unstable distal radius fractures with volar locking plates that the volar plating system appears to provide effective fixation in such cases [13]. Chan BK et al. and McKenna J et al. have shown in their study that the external fixation yielded good results in the management of unstable intraarticular fractures of the distal radius. However, external fixation only is usually not sufficient to address articular incongruence [14-15]. Open reduction and plating have been previously compared with external fixation supplemented with K wiring by quite a few authors. Cui Z et al. conducted a meta-analysis of 738 patients comparing the outcome of internal versus external fixation in intraarticular fracture of the distal radius and concluded that the internal fixation group had a comparatively very low incidence of postop complications with a better clinical outcome and disabilities of the arm, shoulder, and hand (DASH) score at six weeks, with similar results at three months and 12 months postop and supported internal fixation over external fixation [6]. Fu Q et al. conducted a meta-analysis of nine published randomized controlled trials (RCTs) with 776 patients of distal radius fractures treated with either a volar locking plate or external fixation and concluded that volar plating gives better clinical results in the early postop period with better DASH scores even at 12 months follow-up and hence supported the use of volar plating for the management of distal radius fractures [16].

On the other hand, Wei DH et al. and Karantana A et al. found internal fixation and external fixation to have comparable outcomes. Wei DH et al. in their meta-analysis of RCTs found that internal fixation yielded a significantly better functional outcome, anatomical restoration, and forearm supination, but external fixation resulted in better grip strength and wrist flexion and concluded external fixation to be a viable surgical alternative [17-18]. Karantana A et al. conducted a Level 1 RCT to compare the outcome of both techniques and concluded that volar plating leads to quicker recovery of function and better anatomical reduction and power of grip in the early postop period but no functional advantage can be shown beyond 12 weeks as compared to external fixation. Hence, only early recovery in functions may be of benefit to some patients [18]. Shukla et al, Kreder et al.and Saving et al. found the use of external fixation to be superior to internal fixation [1,19-20]. Kreder et al. had conducted an RCT for displaced intraarticular fractures of the distal radius and found that during the two-year follow-up, patients undergoing indirect reduction and external fixation had a more rapid return to function and were found to have a better functional outcome as compared to the internal fixation group, provided articular step and gap deformity were minimized [19]. Shukla et al. in their Level 4 prospective RCT in 110 cases of Cooney’s Type 4 displaced intraarticular fracture of the distal radius observed that the group with an external fixator achieved a superior outcome than the volar locking plate group for movements around the wrist joint, grip power, and final outcome. Further, no difference could be seen in pain and activity in both operative technique groups and concluded that fixation with an external fixator is superior to open reduction internal fixation (ORIF) with volar-locked plating one-year postop [1]. Saving et al. in an RCT of 118 patients of unstable distal radius fracture treated with a volar locking plate or an external fixator found comparable functional results in both groups with a higher incidence of arthrosis and reoperation rates in the volar locking group [20]. Kapoor et al. concluded in their RCT on the displaced intraarticular fractures of the distal radius that cases treated with internal fixation were least likely to develop articular complications due to better restoration of anatomy. However, severely comminuted fractures may present with a poorer functional result due to unstable fixation by locking plates [21]. In such cases, external fixation may lead to superior results by better maintaining radial length by means of sustained traction according to the principle of ligamentotaxis. External fixation-related complications could be easily minimized with meticulous pin insertion and proper postop care. Rogachefsky et al. have advocated the use of the combined approach of open reduction followed by internal and external fixation for severely comminuted AO type C fractures, which provided a satisfactory restoration of anatomy and functional outcome [22].

Our study shows no statistically significant difference in pain score, functional score, and grip strength at the three months' follow-up in both groups but the internal fixation group is at an obvious advantage in the postop period of around three months in terms of the ROM score over the external fixation group and the difference was narrowed by six months of follow-up likely due to the early beginning of wrist ROM physiotherapy as tolerated by the patient as compared to the external fixator group who had to wait for six weeks for the removal of apparatus to start physiotherapy. At the six months' follow-up, the grip strength score of the fixator group is significantly better than the volar plating group may be due to the early starting of grip strengthening exercises started in the fixator group, which were aided by the stabilization of the wrist by an external fixator in the immediate postop period as compared to the plating group who faced surgical site pain and thus delayed grip strength exercises. However, we observed a significant increase in the individual scores of both groups over the course of physiotherapy by the six months' follow-up and more so in the external fixator group so it was on par with the volar plating group in total score according to the Green O’Brien system and the difference in both groups is statistically insignificant at the six months' follow-up. Hattori Y et al. also did not find any statistically significant correlation among groups treated with either plating or external fixation and evaluated using Green & O’Brien scoring [23]. Similar results were seen in a study conducted by Germaine GQ et al., where the Green & O’ Brien score of plating and the fixator group at 12 months was 73 and 74 (p-value=0.76), respectively, and at 24 months were 81 and 80 (p-value=0.85), respectively [24]. These observations are comparable to the final results of our study.

A subanalysis of the data also points out that closed reduction and external fixation of extensively comminuted AO type C2 and C3 fractures have the advantage of shorter operative time and comparable functional outcome when compared to volar plating.

The merits of the study were the randomization of patients, a relatively homogenous cohort in both groups, and the blinded analysis of data. The limitations of our study include small sample size, observer bias, the limited follow-up period of six months, the use of a strict clinical scoring system that focusses heavily on ROM and grip strength.

## Conclusions

Both fixation techniques seem to apply sufficient stabilization to restore function and retain anatomy after an intraarticular fracture of the distal radius but volar locking plates have certain advantages over external fixators in the early postop period in terms of a faster return to the normal daily routine, better early ROM, and better patient tolerance of hardware. Since the external fixator is able to satisfactorily maintain the reduction of severely comminuted (C2, C3) fractures by means of ligamentotaxis, which may sometimes prove to be difficult to address with volar locking plates due to the highly variable fracture anatomy and still give a comparable functional outcome, external fixators can be safely considered for the management of comminuted intraarticular fractures of the distal end radius.
